# Synergistic effect of the commonest residual risk factors, remnant cholesterol, lipoprotein(a), and inflammation, on prognosis of statin-treated patients with chronic coronary syndrome

**DOI:** 10.1186/s12967-022-03448-x

**Published:** 2022-05-26

**Authors:** Hui-Hui Liu, Yuan-Lin Guo, Cheng-Gang Zhu, Na-Qiong Wu, Ying Gao, Rui-Xia Xu, Qian Dong, Jie Qian, Ke-Fei Dou, Jian-Jun Li

**Affiliations:** Cardiometabolic Center, State Key Laboratory of Cardiovascular Disease, Fuwai Hospital, National Center for Cardiovascular Diseases, National Clinical Research Center for Cardiovascular Diseases, Chinese Academy of Medical Sciences and Peking Union Medical College, No. 167 BeiLiShi Road, XiCheng District, Beijing, 100037 China

**Keywords:** Remnant cholesterol, Lipoprotein(a), High-sensitivity C-reactive protein, Prognosis, Chronic coronary syndrome

## Abstract

**Background:**

Currently, remnant cholesterol (RC), lipoprotein(a) [Lp(a)], and inflammation are considered the principal residual cardiovascular risk (RCVR) factors. This study sought to evaluate the combined impact of RC, Lp(a), and inflammation on prognosis of statin-treated patients with chronic coronary syndrome (CCS), which has not been investigated.

**Methods:**

A total of 6839 patients with CCS were consecutively enrolled. Baseline RC, Lp(a), and high-sensitivity C-reactive protein (hsCRP) concentrations were measured and their medians were used for categorizations. All patients were followed for the major adverse cardiovascular events (MACEs), including cardiovascular death, non-fatal myocardial infarction, and stroke. The individual and combined effects of RC, Lp(a), and hsCRP on MACEs were examined and stratification analysis according to low-density lipoprotein cholesterol (LDL-C) was performed.

**Results:**

Over an average of 54.93 ± 18.59 months follow-up, 462 MACEs were recorded. Multivariate Cox analysis showed that elevated RC and Lp(a) levels were significantly associated with an increased risk of MACEs, while high hsCRP levels were related to a slightly but non-significantly increased MACEs risk. Moreover, when participants were subgrouped according to RC, Lp(a), and hsCRP levels together, only High RC-High Lp(a)-High hsCRP group had significantly higher risk of MACEs [hazard ratio (HR) 1.99, 95% confidence interval (CI) 1.15–3.47] compared with the reference group (Low RC-Low Lp(a)-Low hsCRP), especially in patients with LDL-C < 2.6 mmol/L.

**Conclusions:**

The combination of elevated levels of RC, Lp(a), and hsCRP potentiated the adverse effect on MACEs among statin-treated patients with CCS, suggesting that multiple RCVR factors assessment may be a better strategy to improve stratification in very-high risk population.

**Supplementary Information:**

The online version contains supplementary material available at 10.1186/s12967-022-03448-x.

## Introduction

In spite of significant advances in the understanding, prevention, detection, and treatment of atherosclerotic cardiovascular disease (ASCVD), it remains the leading cause of death worldwide [1]. The central, modifiable causal risk factor of the development of ASCVD is dyslipidemia, particularly increased low-density lipoprotein cholesterol (LDL-C) levels [2]. Thus, current guidelines and consensuses have focused on decreasing LDL-C levels with statin, nonstatin, or combination therapies for primary and secondary prevention of ASCVD events [2]. However, patients continue to suffer from cardiovascular events frequently despite being treated effectively with lipid-lowering drugs including intensive statins and even if the LDL-C target is achieved, which is called residual cardiovascular risk (RCVR) [3, 4]. Therefore, there has been a great effort to identify and manage major determinants of RCVR.

Beyond traditional risk factors addressed in usual clinical care [5], residual atherogenic lipoproteins, especially triglyceride-rich lipoproteins (TRLs) and lipoprotein(a) [Lp(a)], and inflammation are suggested to play a critical role in driving RCVR after LDL-C lowering and have been paid great attention in cardiovascular field [2, 6, 7]. Remnant cholesterol (RC) is the cholesterol content of TRLs [8]. There is mounting evidence that elevated RC concentration is causally related to ASCVD risk independent of LDL-C levels [8–12]. While Lp(a) is a heterogenous glycoprotein, which is an apoB100 containing lipoprotein covalently bound to apolipoprotein(a) [13]. Numerous studies including ours have demonstrated the significance of Lp(a) in the development and progression of ASCVD [14–21], even in individuals with LDL-C levels < 1.8 mmol/L [22, 23]. In regard to the residual inflammatory risk, previous studies have indicated that a low systemic inflammation burden, as determined by the level of high-sensitivity C-reactive protein (hsCRP), is associated with a better prognosis in statin-treated patients [24–26]. Inflammation has been suggested to be an important treatment target for the prevention of ASCVD events in high-risk patients [5, 26, 27].

Interestingly, it is indicated that elevated concentration of RC is causally associated with low-grade inflammation [28, 29], and the link of Lp(a) to ASCVD may partially be driven by its pro-inflammatory effects [16]. Based on the evidence regarding the relation of RC, Lp(a), and inflammation to the residual risk following LDL-C lowering therapy and the close relationships among them, we hypothesized that there may be synergetic effects of these three RCVR factors on clinical outcomes in statin-treated patients with ASCVD. Thus, we performed this study to investigate the separate and combined prognostic value of RC, Lp(a), and hsCRP in a large cohort of statin-treated patients with chronic coronary syndrome (CCS).

## Methods

The data that support the findings of this study are available from the corresponding author on reasonable request.

### Study design and population

This study complied with the Declaration of Helsinki and Title 45, US Code of Federal Regulations, Part 46, Protection of Human Subjects, Revised November 13, 2001, effective December 13, 2001.

**Fig. 1:**
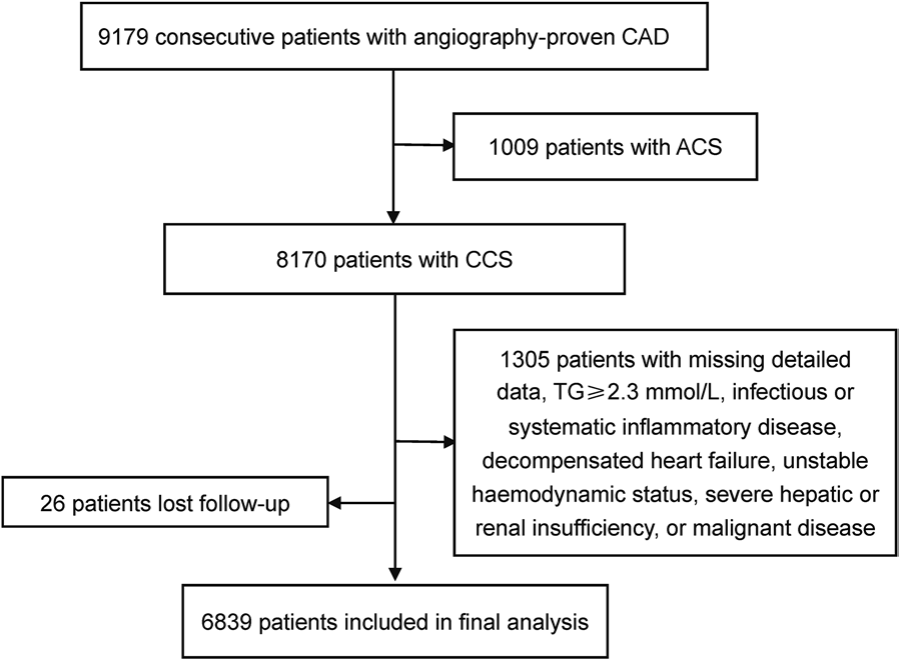
Flowchart illustrating study population. *ACS* acute coronary syndrome, *CAD* coronary artery disease, *CCS* chronic coronary syndrome, *TG* triglyceride

From March 2011 to July 2017, 9179 Chinese patients with angiography-proven coronary artery disease (CAD) were consecutively recruited from Fuwai hospital. CAD was defined according to the evidence of 1 or more of the following: angiographic evidence of at least 50% occlusion of 1 or more coronary arteries or history of percutaneous coronary intervention (PCI)/coronary artery bypass grafting (CABG). Based on elevated myocardial enzyme levels, typical electrocardiogram changes, positive findings by angiography and medical history, 1009 patients with acute coronary syndrome (ACS) were excluded. Furthermore, 1305 patients were excluded due to missing detailed laboratory data, elevated triglyceride (TG) levels (≥ 2.3 mmol/L), uncontrolled decompensated heart failure, unstable hemodynamic status, thyroid dysfunction, infectious or systematic inflammatory diseases, severe hepatic and/or renal insufficiency, and malignant diseases. In addition, 26 patients were lost to follow-up. Finally, 6839 patients with CCS were enrolled into the study (Fig. 1). All patients were categorized according to the medians of RC (0.44 mmol/L), Lp(a) (15.80 mg/dL), and hsCRP (1.31 mg/L) levels, and then by the three indicators together. All enrolled patients were prescribed statin-based therapy for secondary prevention of CAD.

### Biochemical analysis

Blood samples were collected from each patient after at least 12-h fasting in the morning. According to previous studies [9–11], RC was calculated as total cholesterol (TC) minus LDL-C minus high-density lipoprotein cholesterol (HDL-C). The LDL-C level was measured by a selective solubilization method (Low Density Lipid Cholesterol Test Kit, Kyowa Medex, Tokyo, Japan). The HDL-C concentration was determined by a homogeneous method (Determiner L HDL, Kyowa Medex, Tokyo, Japan). TC and TG levels were analyzed by enzymatic assay. As stated in our previous studies [18, 21], Lp(a) concentration was determined by immunoturbidimetry method [LASAY Lp(a) auto, SHIMA Laboratories Co., Ltd] with a normal value of < 30 mg/dL and a Lp(a) protein validated standard was used to calibrate the examination. The level of hsCRP was measured by immunoturbidimetry method (Beckmann Assay 360, Bera, Calif., USA).

### Clinical assessment

Baseline information on demographic factors, personal health habits, medical history, and medication use were collected from each patient by professional cardiologist. The traditional risk factors were defined according to our previous studies [20, 30]. Hypertension was diagnosed by a self-reported hypertension and currently taking antihypertensive drugs, or recorded systolic blood pressure (SBP) ≥ 140 mmHg and/or diastolic blood pressure (DBP) ≥ 90 mmHg for three or more consecutive times. Diabetes mellitus (DM) was defined by fasting plasma glucose ≥ 7.0 mmol/L or the 2-h plasma glucose of the oral glucose tolerance test ≥ 11.1 mmol/L or currently using hypoglycaemic drugs or insulin.

### Follow-up

All participants were actively followed-up at 6-month intervals via clinical visits and/or telephone contacts until July 2019 by well-trained cardiologists or nurses. The major adverse cardiovascular events (MACEs) included cardiovascular death, non-fatal myocardial infarction (MI) and stroke. Cardiovascular death indicated death mainly caused by acute MI, congestive heart failure, malignant arrhythmia, and other structural or functional cardiac diseases. Non-fatal MI was defined as elevated cardiac troponins accompanied by typical chest pain or typical electrocardiogram serial changes. Stroke was diagnosed by persistent neurological dysfunction with documentation of acute cerebral infarction on computed tomography and/or magnetic resonance imaging. Three experienced cardiologists who were blinded to the data classified the events independently.

### Statistical analysis

Continuous variables are expressed as mean ± SD or median (interquartile range) as appropriate and categorical variables are presented as number (percentage). Differences of variables were compared by student’s t-test, nonparametric test, or χ^2^-test where appropriate. The cumulative event-free survival rates among groups were estimated by the Kaplan–Meier analysis and analyzed by the log-rank test. Cox proportional hazard models were performed to calculate the hazard ratios (HRs) and 95% confidence intervals (CIs). Restricted cubic spline (RCS) adjusted for age and sex was implemented to evaluate linearity assumptions of the association of RC, Lp(a), and hsCRP with MACEs. Additionally, correlation analyses and interactive analyses for predicting the risk of MACEs were performed among the three indicators. Stratification analysis was conducted to further clarify whether the association of RC, Lp(a), and hsCRP with MACEs would be altered by LDL-C levels. For all analyses, two tailed *p*-values < 0.05 were considered statistically significant. The statistical analyses were performed with SPSS version 24.0 software (SPSS Inc., Chicago, IL, USA), R language version 3.5.2 (Feather Spray), and STATA version 15.1 (StataCorp, College Station, TX, USA).

### Role of the funding source

The funding sources for the study played no role in the study’s design, conduct, and reporting.

## Results

### Baseline characteristics

The mean age of total subjects was 58.1 years and 72.4% of them were males. During an average of 54.93 ± 18.59 months follow-up, 462 MACEs (197 cardiovascular deaths, 94 non-fatal MIs, and 171 strokes) were recorded, representing 14.8 events per 1000 person-years. As shown in Table 1, compared with event-free patients, those who suffered from MACEs had significantly higher levels of RC, Lp(a), and hsCRP. Additionally, patients with incident MACEs were slightly older and had a higher proportion of hypertension, DM, and prior MI, higher levels of SBP, glycosylated hemoglobin, and creatinine, but lower levels of DBP and left ventricular ejection fraction (LVEF), than those without MACEs. When it comes to the baseline statin use, patients in MACEs group were less likely to take statins at admission and had a relatively higher proportion of simvastatin use at admission and a lower proportion of pitavastatin use on discharge, compared with those in event-free group. However, statin intensities at admission or on discharge and the proportion of statin use on discharge were comparable between event and event-free groups. Meanwhile, there was no significant difference respect to the other drugs use between two groups (see Additional file 1: Table S1).

**Table 1:** Baseline characteristics of patients with and without MACEs

Variables	Overall subjects (n = 6839)	MACEs (n = 462)	Without MACEs (n = 6377)	*p* value
Age, years	58.1 ± 10.7	63.1 ± 10.4	57.8 ± 10.6	< 0.001
Male, n (%)	4949 (72.4)	332 (71.9)	4617 (72.4)	0.836
Hypertension, n (%)	4212 (61.6)	316 (68.3)	3896 (61.1)	0.009
Diabetes, n (%)	1843 (27.0)	166 (36.0)	1677 (26.3)	< 0.001
Smoking status				0.184
Never smoker, n (%)	3120 (45.6)	210 (45.5)	2910 (45.6)	
Former smoker, n (%)	929 (13.6)	77 (16.7)	852 (13.4)	
Current smoker, n (%)	2790 (40.8)	176 (38.0)	2614 (41.0)	
Prior MI, n (%)	2053 (30.0)	191 (41.3)	1862 (29.2)	< 0.001
Prior RV, n (%)	1998 (29.2)	155 (33.5)	1843 (28.9)	0.073
Family history of CAD, n (%)	926 (13.5)	59 (12.8)	867 (13.6)	0.707
BMI, kg/m^2^	25.67 ± 3.19	25.50 ± 3.28	25.68 ± 3.18	0.309
SBP, mmHg	127 ± 17	129 ± 19	126 ± 17	0.019
DBP, mmHg	77 ± 11	76 ± 11	77 ± 11	0.025
LVEF, %	63.39 ± 8.15	59.84 ± 11.26	63.57 ± 7.92	< 0.001
Biochemical parameters				
FPG, mmol/L	5.75 ± 1.65	5.86 ± 1.87	5.75 ± 1.63	0.287
HbA1c, %	6.27 ± 1.06	6.58 ± 1.24	6.26 ± 1.04	< 0.001
TC, mmol/L	3.96 ± 1.03	4.00 ± 1.11	3.95 ± 1.03	0.469
HDL-C, mmol/L	1.08 ± 0.29	1.08 ± 0.30	1.08 ± 0.29	0.894
LDL-C, mmol/L	2.44 ± 0.92	2.44 ± 1.00	2.44 ± 0.92	0.991
TG, mmol/L	1.33 (1.02–1.69)	1.31 (0.99–1.73)	1.33 (1.02–1.69)	0.878
Lp(a), mg/dL	15.80 (7.11–37.87)	19.74 (8.93–43.75)	15.68 (7.05–37.61)	0.008
RC, mmol/L	0.44 ± 0.24	0.48 ± 0.23	0.43 ± 0.24	0.001
HsCRP, mg/L	1.31 (0.71–2.77)	1.62 (0.89–3.63)	1.29 (0.71–2.72)	< 0.001
Creatinine, µmol/L	77.91 ± 18.57	80.84 ± 18.78	77.76 ± 18.55	0.003

Continuous values are summarized as mean ± SD, median (interquartile range) and categorical variables as number (percentage)

*BMI* body mass index, *CAD* coronary artery disease, *DBP* diastolic blood pressure, *FPG* fasting plasma glucose, *HbA1c* glycosylated hemoglobin, *HDL-C* high-density lipoprotein cholesterol, *HsCRP* high-sensitivity C-reactive protein, *LVEF* left ventricular ejection fraction, *LDL-C* low-density lipoprotein cholesterol, *Lp(a)* lipoprotein(a), *MACEs* major adverse cardiovascular events, *MI* myocardial infarction, *RV* revascularization, *RC* remnant cholesterol, *SBP* systolic blood pressure, *TC* total cholesterol, *TG* triglyceride

### Association of RC, Lp(a), and hsCRP with MACEs risk

**Fig. 2:**
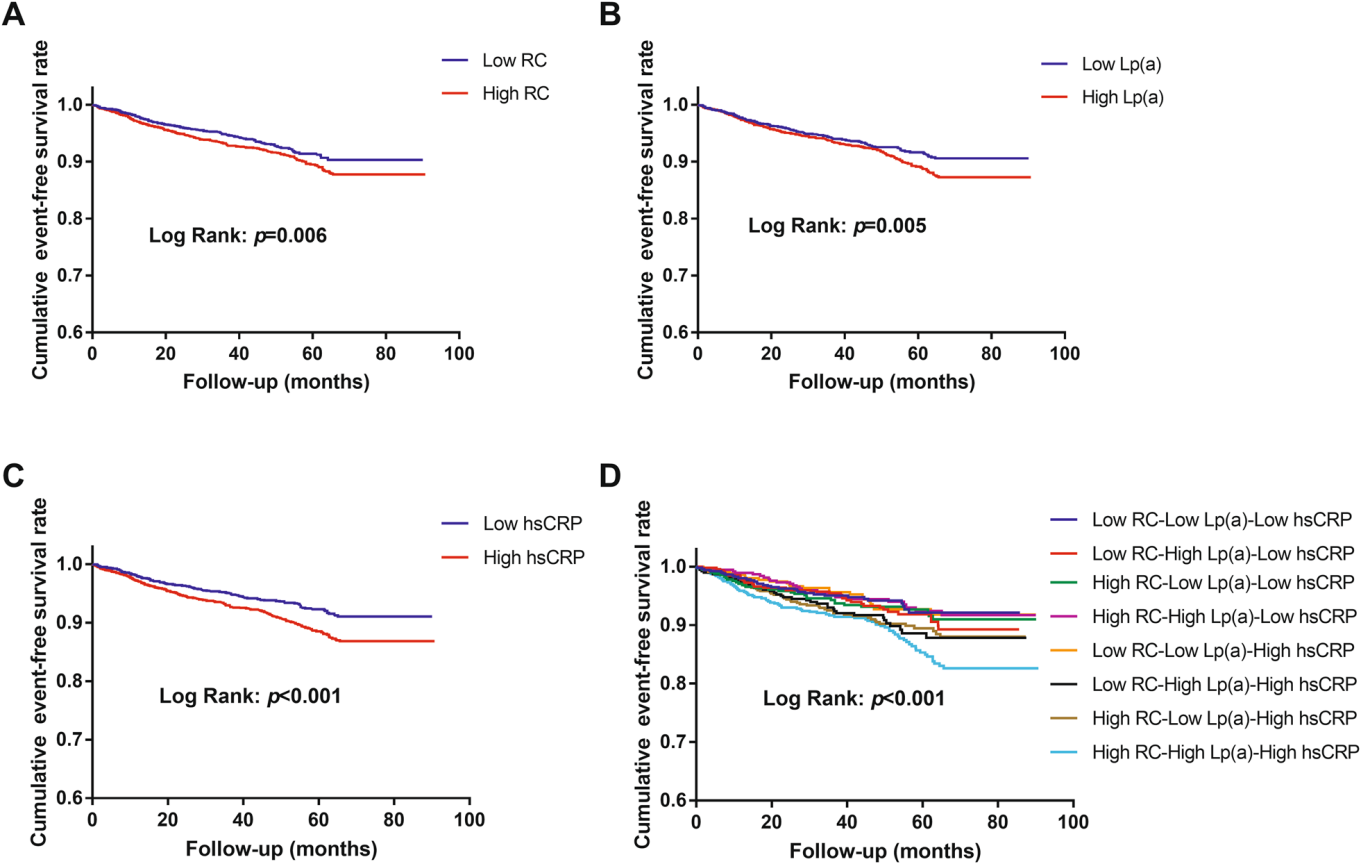
The cumulative event-free survival analyses according to RC, Lp(a), and/or hsCRP. **A** RC; **B** Lp(a); **C** hsCRP; **D** RC, Lp(a) and hsCRP. *hsCRP* high-sensitivity C-reactive protein, *Lp(a)* lipoprotein(a), *RC* remnant cholesterol

As shown in Fig. 2, the Kaplan–Meier analysis showed that subjects with high levels of RC, Lp(a) or hsCRP (≥ median respectively) had significantly lower event-free survival rates compared to those with low levels of them (< median respectively; all *p* < 0.05). When subjects were categorized according to RC, Lp(a) and hsCRP together, the Kaplan–Meier analysis showed that only those with high levels of these three indicators simultaneously had significantly lower event-free survival rates compared with participants in the reference group (Low RC-Low Lp(a)-Low hsCRP; *p* = 0.003).

**Fig. 3:**
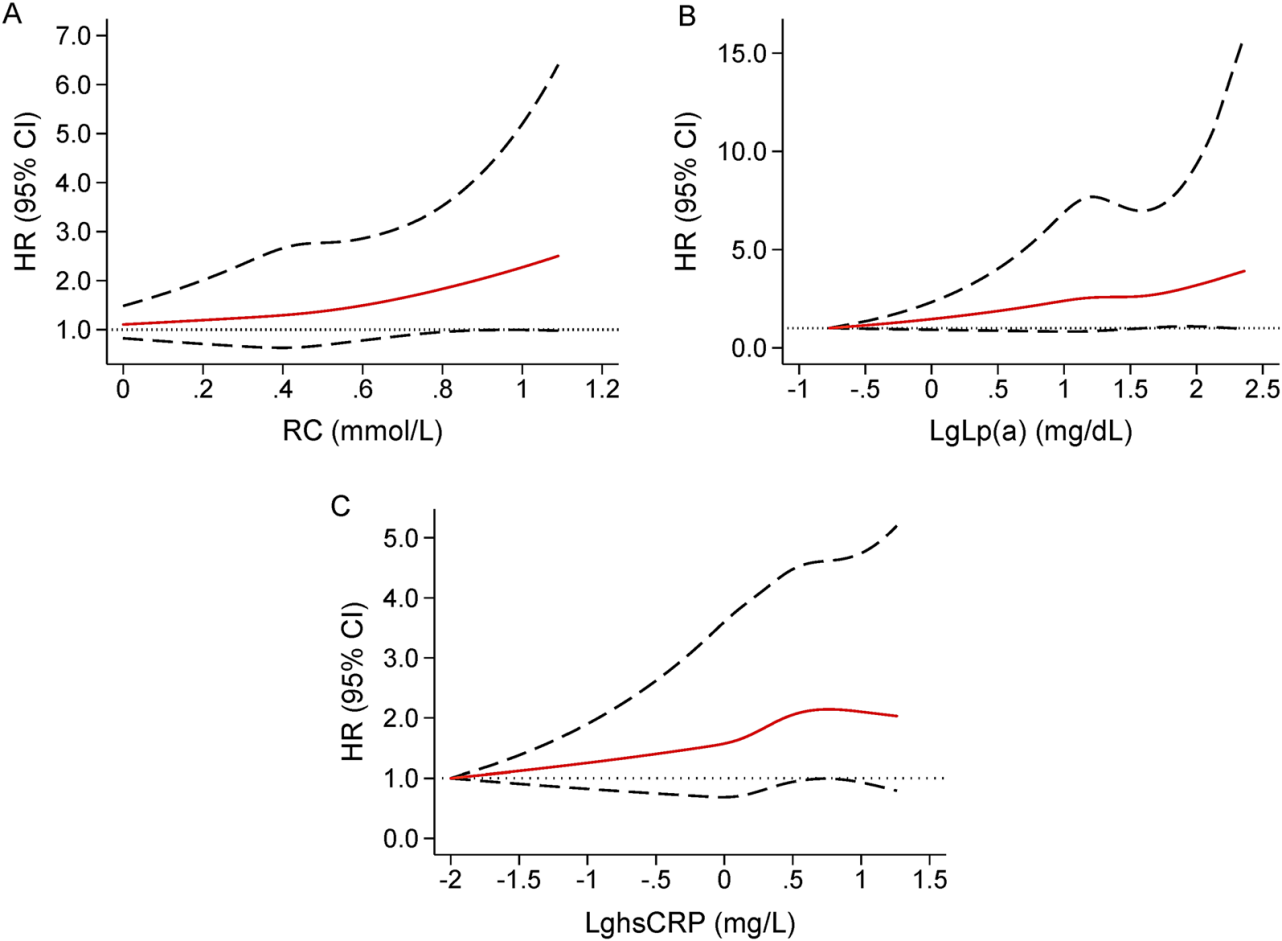
Age- and sex-adjusted RCS plot of the association of RC, Lp(a), and hsCRP with MACEs. **A** RC, **B** Lp(a); **C** hsCRP. *LgLp(a)* log-transformed lipoprotein(a), *LghsCRP* log-transformed high-sensitivity C-reactive protein, *MACEs* major adverse cardiovascular events, *RCS* restricted cubic spline, *RC* remnant cholesterol

Furthermore, after adjusted for potential covariates, the significant association of high levels of RC (HR 1.47, 95% CI 1.05–2.05) and Lp(a) (HR 1.40, 95% CI 1.05–1.86) with MACEs still existed. Per 1-SD increase of RC and LgLp(a) were associated with a respective 26% and 24% increase of the risk of MACEs. In addition, elevated hsCRP levels were related to a slight but non-significant increase of the MACEs risk (HR 1.27, 95% CI 0.95–1.70; Table 2). As shown in Fig. 3, RCS showed a strong trend toward non-linear positive association of RC, Lp(a), and hsCRP with MACEs in our patients. Moreover, among subgroups divided by RC, Lp(a), and hsCRP together, multivariate Cox regression analysis showed that compared with the reference group (Low RC-Low Lp(a)-Low hsCRP), High RC-High Lp(a)-High hsCRP group had a 1.99-fold (95% CI 1.15–3.47) higher risk of MACEs, while the other six groups had no significantly increased MACEs risk (all *p* > 0.05; Table 2). In stratification analysis according to LDL-C levels, we observed similar results among subjects with LDL-C levels < 2.6 mmol/L, that is elevated RC (HR 1.39, 95% CI 1.04–1.87) and Lp(a) (HR 1.53, 95% CI 1.08–2.17) levels were significantly associated with a higher risk of MACEs, while high levels of hsCRP (HR 1.28, 95% CI 0.99–1.65) were related to a slightly but non-significantly increased risk of MACEs. Additionally, when categorizing according to the three indicators together, only patients had higher levels of RC, Lp(a), and hsCRP simultaneously had a significantly higher risk of MACEs (HR 1.81, 95% CI 1.14–2.89) compared with those with lower levels of them. However, in patients with LDL-C levels ≥ 2.6 mmol/L, we observed no significant associations between individuals or the combination of elevated RC, Lp(a), and hsCRP concentrations and the risk of MACEs (see Additional file 1: Table S2).

**Table 2:** Cox regression analyses of RC, Lp(a), and hsCRP levels for predicting MACEs

Category	Model 1 HR (95% CI)	Model 2 HR (95% CI)
RC		
Low RC	1.00 (reference)	1.00 (reference)
High RC	1.29 (1.03–1.61)*	1.47 (1.05–2.05)*
Per 1-SD increase of RC	1.17 (1.05–1.32)^†^	1.26 (1.06–1.50)*
Lp(a)		
Low Lp(a)	1.00 (reference)	1.00 (reference)
High Lp(a)	1.26 (1.01–1.57)*	1.40 (1.05–1.86)*
Per 1-SD increase of LgLp(a)	1.16 (1.04–1.30)*	1.24 (1.07–1.44)*
HsCRP		
Low hsCRP	1.00 (reference)	1.00 (reference)
High hsCRP	1.39 (1.11–1.75)^†^	1.27 (0.95–1.70)
Per 1-SD increase of LghsCRP	1.17 (1.04–1.32)^†^	1.09 (0.94–1.26)
RC, Lp(a), and hsCRP		
Low RC-Low Lp(a)-Low hsCRP	1.00 (reference)	1.00 (reference)
Low RC-High Lp(a)-Low hsCRP	1.22 (0.76–1.96)	1.09 (0.59–2.01)
High RC-Low Lp(a)-Low hsCRP	1.19 (0.73–1.95)	1.00 (0.52–1.93)
High RC-High Lp(a)-Low hsCRP	1.06 (0.63–1.80)	1.39 (0.74–2.61)
Low RC-Low Lp(a)-High hsCRP	0.97 (0.57–1.67)	0.72 (0.36–1.46)
Low RC-High Lp(a)-High hsCRP	1.45 (0.90–2.33)	1.30 (0.71–2.37)
High RC-Low Lp(a)-High hsCRP	1.46 (0.94–2.27)	1.46 (0.82–2.61)
High RC-High Lp(a)-High hsCRP	1.95 (1.29–2.95)^†^	1.99 (1.15–3.47)*

Model 1 adjusted for age and sex; Model 2 adjusted for age, sex, smoking status, prior myocardial infarction, hypertension, diabetes, left ventricular ejection fraction, triglyceride, low-density lipoprotein cholesterol, high-density lipoprotein cholesterol; creatinine, statin use and types at admission, and statin types on discharge

*HsCRP* high-sensitivity C-reactive protein, *Lp(a)* lipoprotein(a), *LgLp(a)* log-transformed Lp(a), *LghsCRP* log-transformed hsCRP, *MACEs* major adverse cardiovascular events, *RC* remnant cholesterol

*p < 0.05; ^†^*p* < 0.01

The correlation analysis showed that there was a significant and positive association between any two of RC, Lp(a), and hsCRP (all *p* < 0.05; see Additional file 1: Table S3). Moreover, the interactive analyses showed that there were synergistic relationships between RC and Lp(a) [Relative Excess Risk of Interaction (RERI): 1.72, 95% CI 0.51–3.94; Attributable Proportion (AP): 0.49, 95% CI 0.27–0.71; Synergy Index (SI): 3.17, 95% CI 2.25–4.46; all *p* < 0.05], RC and hsCRP (RERI: 1.06, 95% CI 0.18–2.29; AP: 0.46, 95% CI 0.29–0.64; SI: 5.68, 95% CI 1.16–27.75; all *p* < 0.05), and Lp(a) and hsCRP (RERI: 1.30, 95% CI 0.27–2.87; AP: 0.48, 95% CI 0.29–0.67; SI: 4.33, 95% CI 2.23–8.42; all *p* < 0.05).

## Discussion

The holistic view of multiple risk factors maybe a wiser way for risk stratification and management of ASCVD. In this prospective analysis, we observed that elevated RC and Lp(a) levels were associated with a respective 1.47-fold and 1.40-fold increased risk of MACEs among statin-treated patients with CCS, while high levels of hsCRP were related to a slightly but non-significantly increased MACEs risk. When participants were categorized according to RC, Lp(a), and hsCRP levels together, only patients with high levels of them three had a significant 1.99-fold higher risk of MACEs compared with those in the reference group (Low RC-Low Lp(a)-Low hsCRP). Moreover, interaction analyses showed that there was a synergistic relationship between any two of RC, Lp(a), and hsCRP for predicting the risk of MACEs. These findings suggested that the combination of RC, Lp(a), and hsCRP was of great importance for risk stratification in statin-treated patients with CCS.

Statin is considered as the treatment of choice for reducing plasma cholesterols, and is regarded as a corner-stone of primary and secondary prevention of ASCVD by most guidelines [31]. Unlike the recommendation of high-intensity statins in western populations, moderate-intensity statins are recommended as the initial treatment in China due to the relatively lower levels of plasma cholesterols and greater sensitivity to statins of Chinese population [32, 33]. Thus, in the present study, 93.9% of patients were prescribed moderate-intensity statins on discharge, while only 4.5% took high-intensity statins. Recently, antibodies and synthetic small interfering RNA (siRNA) targeting the serine-protease proprotein convertase subtilisin–kexin type-9 (PCSK9), a physiological regulator of LDL-C, have increasingly been used in patients with high ASCVD risk and brought about a further effective decline of LDL-C concentrations with good security [34, 35]. However, a relatively high proportion of patients remain at high risk of cardiovascular events in spite of achieving guideline-recommended treatment targets [36, 37]. Thus, RCVR in the current era poses new challenges on the prevention of ASCVD events despite managing traditional risk factors, including hypertension, DM, and unhealthy lifestyle [5, 38, 39]. Targeting the RCVR has been the primary goal of a large number of pharmacotherapies and has attained a measure of success [36]. Nonetheless, incomplete understanding of the specific mechanisms and the lack of efficient tools to precisely evaluate the RCVR contributed to the relatively high residual risk under optimal medical therapy, which has aroused substantial concern and evoked great efforts to identify most important contributors and more suitable targets to further reduce the RCVR [36, 37].

In recent years, emerging evidence suggests the crucial significance of residual atherogenic lipoprotein burden in causing ASCVD events [2, 6, 37]. Among non-LDL-C lipid parameters, RC and Lp(a) are two well-demonstrated factors inducing RCVR [2]. Elevated RC levels were shown to be associated with a high cardiovascular risk in both primary and secondary prevention cohorts [8–10, 12, 40]. Mendelian randomization study suggested that per 1.0 mmol/L increase of RC was associated with a 2.8-fold causal risk of ischemic heart disease, independent of LDL-C or HDL-C levels [11]. Moreover, a post hoc analysis from TNT trial showed that the reduction of RC levels with statins resulted in a significant lower risk of MACE beyond the reduction in LDL-C concentrations [10]. As to Lp(a), it may mediate atherogenicity via its LDL moiety and induce prothrombotic effect through the plasminogen-like apolipoprotein(a) component [16]. In addition, it also potentially exerts proinflammatory properties via accumulation of oxidized phospholipids [16]. There has been considerable evidence that elevated baseline and on-statin Lp(a) concentrations are independently related to the risk of ASCVD events [14–21, 41]. It has been reported that Lp(a) becomes a more potent predictor of residual risk when LDL-mediated risk is diminished with statins, since statin cannot reduce Lp(a)-mediated risk [41]. Furthermore, the FOURIER and ODYSSEY OUTCOMES trials have suggested that elevated Lp(a) level remains a significant risk factor for ASCVD events in patients with on-treatment LDL-C levels < 1.3 mmol/L [42, 43]. All the above evidence reveals that RC and Lp(a) are two crucial and promising non-traditional RCVR factors. Undoubtedly, our data further confirmed that elevated RC and Lp(a) levels were significantly related to the risk of MACEs in statin-treated patients with CCS after adjusting for traditional risk factors including LDL-C. Stratification analysis according to LDL-C levels showed that the trends of relationship of RC and Lp(a) to the MACEs risk were consistent in both strata. However, in patients with LDL-C levels ≥ 2.6 mmol/L, elevated RC and Lp(a) were associated with a slightly but non-significantly increased risk for MACEs, which may be due to the small sample size.

In addition, residual inflammatory risk after statin and other lipid-lowering drugs has also been greatly appreciated in recent years [7]. From the earlier JUPITER [24] to the recently published FOURIER [25] and a post hoc analysis of the SPIRE trials [44], inflammation plays a significant role in the occurrence of cardiovascular events independent of circulating LDL-C levels. HsCRP, a most common used biomarker reflecting systemic inflammation, was reported to be an important and independent predictor of the incidence and prognosis of ASCVD [25, 44–46]. The benefits of anti-inflammatory effects of statins have already been proved [47–49]. Furthermore, recent trials of anti-inflammatory therapies targeted at specific inflammatory pathways have further demonstrated the efficacy for the prevention of cardiovascular events [26, 27]. Therefore, interfering residual inflammatory risk is necessary in addition to managing LDL-C and residual cholesterol risk [50]. In the present study, elevated hsCRP level was also associated with a higher risk of MACEs, but this was not statistically significant, which may be related to the cut-off value (median) used in this study.

Currently, the importance of composite risk factor evaluation and control for stratifying and reducing ASCVD risk has gained increased attention. It has been reported that when multiple risk factors are present, the increase of risk is often synergistic rather than additive [51]. For example, in the MRFIT (Multiple Risk Factor Intervention Trial) with an average follow-up of 12 years, the coronary mortality for nonsmoking men, with SBP < 120 mmHg and serum TC concentration < 4.7 mmol/L, was 3.1 per 10,000 person-years. For those with SBP over 142 mmHg, it was 13.7; and for those with serum TC level over 6.3 mmol/L, it was 12.2. Moreover, when three risk factors were present together, as in smokers with SBP > 142 mmHg and serum TC concentration > 6.3 mmol/L, the coronary mortality was 62.6 per 10,000 person-years [52]. As stated above, Lp(a) may accelerate the cardiovascular risk through its pro-inflammatory effects. Meanwhile, recent researches indicated that RC was causally associated with low-grade inflammation marked by elevated hsCRP levels [28, 29]. Given the potential close connections among RC, Lp(a), and inflammation and their associations with the RCVR, there may be synergistic effects of them on clinical prognosis in statin-treated patients. To clarify this issue, we performed an analysis categorizing the study population according to RC, Lp(a) and hsCRP levels together and found that the combination of elevated levels of the three indicators indeed greatly worsened the outcomes of statin-treated patients with CCS.

Moreover, compared with patients with low levels of RC, Lp(a), and hsCRP, only those with elevated levels of all them three had a significantly 1.99-fold higher risk of MACEs and this phenomenon mainly existed in patients with LDL-C levels less than 2.6 mmol/L. Positive correlations and synergistic relationships for predicting MACEs risk were observed between any two of these three RCVR factors. The insignificant association of the combination of RC, Lp(a), and hsCRP with MACEs risk in subjects with LDL-C concentrations over 2.6 mmol/L may be limited to the relatively small sample size of this subgroup. In aggregate, our data suggested that a joint assessment of RC, Lp(a), and hsCRP might be a better strategy to further improve RCVR stratification in patients with established CAD under statin therapy.

Nonetheless, this study has several limitations. First, RC concentration was acquired by calculation, but not direct measurement. However, these two kinds of methods show a good correlation and the calculated method used in this study has been adopted in multiple previous studies [9–11]. Second, the concentrations of Lp(a) are obviously different among various ethnicities, which might impact the generalizability of our findings. Third, because of the nature of observational studies, we didn’t have follow-up data of RC, Lp(a), and hsCRP levels, which might provide incremental value for the stratification of MACEs risk. Fourth, the prescribed medications of the patients may be improved based on the update guidelines over the follow-up period, which might influence the outcomes.

## Conclusions

In summary, the present study for the first time indicated that there were synergistic relationships of elevated levels of RC, Lp(a), and hsCRP for predicting MACEs risk and the combination of them could greatly worsen the outcomes of statin-treated patients with CCS, especially in those with LDL-C levels < 2.6 mmol/L. These novel findings suggested that combined evaluation of the major RCVR factors, RC, Lp(a), and inflammation, may be a better strategy for risk stratification in the statin era.

## Supplementary Information

**Additional file 1: Table S1.** Medications at admission and on discharge of patients with and without MACEs. **Table S2.** Cox regression analyses of RC, Lp(a), and hsCRP levels for predicting MACEs according to LDL-C levels. **Table S3.** Pearson correlation analyses of the association among RC, Lp(a), and hsCRP.

## Data Availability

The datasets used and/or analysed during the current study are available from the corresponding author on reasonable request.

## Abbreviations

ASCVD: Atherosclerotic cardiovascular disease
LDL-C: Low-density lipoprotein cholesterol
RCVR: Residual cardiovascular risk
TRLs: Triglyceride-rich lipoproteins
Lp(a): Lipoprotein(a)
RC: Remnant cholesterol
hsCRP: High-sensitivity C-reactive protein
CCS: Chronic coronary syndrome
CAD: Coronary artery disease
MI: Myocardial infarction
PCI: Percutaneous coronary intervention
CABG: Coronary artery bypass grafting
ACS: Acute coronary syndrome
TG: Triglyceride
TC: Total cholesterol
HDL-C: High-density lipoprotein cholesterol
HR: Hazard ratio
CI: Confidence interval
RCS: Restricted cubic spline
SBP: Systolic blood pressure
DBP: Diastolic blood pressure
DM: Diabetes mellitus
MACE: Major adverse cardiovascular event
LVEF: Left ventricular ejection fraction
RERI: Relative Excess Risk of Interaction
AP: Attributable Proportion
SI: Synergy Index
