# Supplementary material for: Synergistic effect of the commonest residual risk factors, remnant cholesterol, lipoprotein(a), and inflammation, on prognosis of statin-treated patients with chronic coronary syndrome
Source: J Transl Med. 2022 May 26;20:243. doi: 10.1186/s12967-022-03448-x (PMC9134647; doi:10.1186/s12967-022-03448-x)
Supplement: Supplementary file 1 — Additional file 1: Table S1. Medications at admission and on discharge of patients with and without MACEs. Table S2. Cox regression analyses of RC, Lp(a), and hsCRP levels for predicting MACEs according to LDL-C levels. Table S3. Pearson correlation analyses of the association among RC, Lp(a), and hsCRP. [file 12967_2022_3448_MOESM1_ESM.docx]

**Table S1.** Medications at admission and on discharge of patients with and without MACEs

| **Variables** | **Overall subjects**  **(n=6839)** | **MACEs**  **(n=462)** | **Without MACEs**  **(n=6377)** | ***p* value** |
| --- | --- | --- | --- | --- |
| Medications at admission |  |  |  |  |
| Statins | 5316 (77.7) | 316 (68.4) | 5000 (78.4) | <0.001 |
| Statin types |  |  |  | 0.036 |
| Atorvastatin, n (%)^*^ | 2793 (52.5) | 158 (50.0) | 2635 (52.7) |  |
| Rosuvastatin, n (%)^*^ | 966 (18.2) | 51 (16.1) | 915 (18.3) |  |
| Simvastatin, n (%)^*^ | 998 (18.8) | 88 (27.8) | 910 (18.2) |  |
| Pitavastatin, n (%)^*^ | 263 (4.9) | 8 (2.5) | 255 (5.1) |  |
| Pravastatin, n (%)^*^ | 174 (3.3) | 4 (1.3) | 170 (3.4) |  |
| Others^†^, n (%)^*^ | 122 (2.3) | 7 (2.2) | 115 (2.3) |  |
| Statin intensity |  |  |  | 0.462 |
| Low-intensity, n (%)^*^ | 368 (6.9) | 18 (5.7) | 350 (7.0) |  |
| Moderate-intensity, n (%)^*^ | 4762 (89.6) | 292 (92.4) | 4470 (89.4) |  |
| High-intensity, n (%)^*^ | 186 (3.5) | 6 (1.9) | 180 (3.6) |  |
| Aspirin, n (%) | 4893 (71.5) | 315 (68.2) | 4578 (71.8) | 0.097 |
| ACEI/ARB, n (%) | 1490 (21.8) | 88 (19.0) | 1402 (22.0) | 0.116 |
| β-blockers, n (%) | 2913 (42.6) | 184 (39.8) | 2729 (42.8) | 0.376 |
| CCB, n (%) | 1354 (19.8) | 79 (17.1) | 1275 (20.0) | 0.320 |
| Medications on discharge |  |  |  |  |
| Statins | 6584 (96.3) | 449 (97.1) | 6135 (96.2) | 0.474 |
| Statin types |  |  |  | 0.025 |
| Atorvastatin, n (%)^*^ | 3394 (51.6) | 233 (52.0) | 3161 (51.5) |  |
| Rosuvastatin, n (%)^*^ | 1384 (21.0) | 113 (25.1) | 1271 (20.7) |  |
| Simvastatin, n (%)^*^ | 58 (0.9) | 2 (0.4) | 56 (0.9) |  |
| Pitavastatin, n (%)^*^ | 1275 (19.4) | 53 (11.9) | 1222 (19.9) |  |
| Pravastatin, n (%)^*^ | 437 (6.6) | 44 (9.7) | 393 (6.4) |  |
| Others^†^, n (%)^*^ | 36 (0.5) | 4 (0.9) | 32 (0.5) |  |
| Statin intensity |  |  |  | 0.169 |
| Low-intensity, n (%)^*^ | 106 (1.6) | 14 (3.1) | 92 (1.5) |  |
| Moderate-intensity, n (%)^*^ | 6184 (93.9) | 417 (92.8) | 5767 (94.0) |  |
| High-intensity, n (%)^*^ | 294 (4.5) | 18 (4.0) | 276 (4.5) |  |
| Aspirin, n (%) | 6607 (96.6) | 447 (96.7) | 6160 (96.6) | 0.942 |
| ACEI/ARB, n (%) | 3069 (44.9) | 225 (48.8) | 2844 (44.6) | 0.201 |
| β-blockers, n (%) | 5276 (77.1) | 366 (79.3) | 4910 (77.0) | 0.322 |
| CCB, n (%) | 2566 (37.5) | 162 (35.0) | 2404 (37.7) | 0.406 |

Categorical variables are summarized as number (percentage). ACEI, angiotensin converting enzyme inhibitor; ARB, angiotensin receptor blocker; CCB, calcium-channel blocker; MACEs, major adverse cardiovascular events.

^*^The proportion of patients with statin use; ^†^Lovastatin or Fluvastatin.

**Table S2.** Cox regression analyses of RC, Lp(a), and hsCRP levels for predicting MACEs according to LDL-C levels

| Category | Adjusted HR (95% CI) | | |  |
| --- | --- | --- | --- | --- |
|  | **LDL-C <2.6 mmol/L**  **(n=4331)** |  | **LDL-C ≥2.6 mmol/L**  **(n=2508)** | |
| RC |  |  |  | |
| Low RC | 1.00 (reference) |  | 1.00 (reference) | |
| High RC | 1.39 (1.04-1.87)^*^ |  | 1.13 (0.76-1.67) | |
| Per 1-SD increase of RC | 1.35 (1.15-1.59)‡ |  | 1.13 (0.93-1.37) | |
| Lp(a) |  |  |  | |
| Low Lp(a) | 1.00 (reference) |  | 1.00 (reference) | |
| High Lp(a) | 1.53 (1.08-2.17)^*^ |  | 1.31 (0.81-2.12) | |
| Per 1-SD increase of LgLp(a) | 1.25 (1.05-1.49)^*^ |  | 1.24 (0.95-1.61) | |
| HsCRP |  |  |  | |
| Low hsCRP | 1.00 (reference) |  | 1.00 (reference) | |
| High hsCRP | 1.28 (0.99-1.65) |  | 1.06 (0.74-1.51) | |
| Per 1-SD increase of LghsCRP | 1.16 (1.02-1.32)^*^ |  | 0.98 (0.82-1.16) | |
| Lp(a), RC and hsCRP |  |  |  | |
| Low RC-Low Lp(a)-Low hsCRP | 1.00 (reference) |  | 1.00 (reference) | |
| Low RC-High Lp(a)-Low hsCRP | 1.01 (0.62-1.66) |  | 1.08 (0.50-2.35) | |
| High RC-Low Lp(a)-Low hsCRP | 0.71 (0.38-1.30) |  | 1.12 (0.49-2.56) | |
| High RC-High Lp(a)-Low hsCRP | 1.09 (0.63-1.90) |  | 1.30 (0.59-2.90) | |
| Low RC-Low Lp(a)-High hsCRP | 0.58 (0.32-1.03) |  | 0.45 (0.17-1.20) | |
| Low RC-High Lp(a)-High hsCRP | 1.16 (0.71-1.87) |  | 1.74 (0.85-3.57) | |
| High RC-Low Lp(a)-High hsCRP | 1.28 (0.78-2.11) |  | 1.29 (0.63-2.64) | |
| High RC-High Lp(a)-High hsCRP | 1.81 (1.14-2.89)^*^ |  | 1.27 (0.63-2.57) | |

hsCRP, high sensitivity C-reactive protein; Lp(a), lipoprotein(a); LDL-C, low-density lipoprotein cholesterol; LgLp(a), log-transformed Lp(a); LghsCRP, log-transformed hsCRP; MACEs, major adverse cardiovascular events; RC, remnant cholesterol. The adjusted Model included age, sex, smoking status, prior myocardial infarction, hypertension, diabetes, left ventricular ejection fraction, triglyceride, high-density lipoprotein cholesterol; creatinine, statin use and types at admission, and statin types on discharge.

^*^*p*<0.05; †, *p*<0.01; ‡, *p*<0.001.

**Table S3.** Pearson correlation analyses of the association among RC, Lp(a), and hsCRP

| **Variable** | **RC** | |  | **LghsCRP** | |
| --- | --- | --- | --- | --- | --- |
|  | **r** | ***p* value** |  | **r** | ***p* value** |
| **RC** | - | - |  | 0.175 | <0.001 |
| **LgLp(a)** | 0.026 | 0.033 |  | 0.067 | <0.001 |

hsCRP, high sensitivity C-reactive protein; Lp(a), lipoprotein(a); LDL-C, low-density lipoprotein cholesterol; LgLp(a), log-transformed Lp(a); LghsCRP, log-transformed hsCRP.
